# The Montreal Medical Journal

**Published:** 1888-10

**Authors:** 


The Montreal *Medical Journal* (formerly the *Canada Medical and Surgical Journal*) has put on a new dress, increased its size to eighty pages, and reduced its subscription price to two dollars per annum. This is, indeed, a radical change, and makes it a model of its class. We wish it all the success it so abundantly deserves.

